# Limited evidence for evolutionarily conserved targeting of long non-coding RNAs by microRNAs

**DOI:** 10.1186/1758-907X-4-4

**Published:** 2013-08-20

**Authors:** Babak Alaei-Mahabadi, Erik Larsson

**Affiliations:** 1 Department of Medical Biochemistry and Cell Biology, Institute of Biomedicine, The Sahlgrenska Academy, University of Gothenburg, SE-405 30, Gothenburg, Sweden

**Keywords:** Long non-coding RNA, lncRNA, microRNA, Comparative genomics

## Abstract

**Background:**

Long non-coding RNAs (lncRNAs) are emerging as important regulators of cell physiology, but it is yet unknown to what extent lncRNAs have evolved to be targeted by microRNAs. Comparative genomics has previously revealed widespread evolutionarily conserved microRNA targeting of protein-coding mRNAs, and here we applied a similar approach to lncRNAs.

**Findings:**

We used a map of putative microRNA target sites in lncRNAs where site conservation was evaluated based on 46 vertebrate species. We compared observed target site frequencies to those obtained with a random model, at variable prediction stringencies. While conserved sites were not present above random expectation in intergenic lncRNAs overall, we observed a marginal over-representation of highly conserved 8-mer sites in a small subset of cytoplasmic lncRNAs (12 sites in 8 lncRNAs at 56% false discovery rate, *P* = 0.10).

**Conclusions:**

Evolutionary conservation in lncRNAs is generally low but patch-wise high, and these patches could, in principle, harbor conserved target sites. However, while our analysis efficiently detected conserved targeting of mRNAs, it provided only limited and marginally significant support for conserved microRNA-lncRNA interactions. We conclude that conserved microRNA-lncRNA interactions could not be reliably detected with our methodology.

## Findings

### Background

While small non-coding RNAs, such as microRNAs, have well-established functions in the cell, long non-coding RNAs (lncRNAs) have only recently started to emerge as widespread regulators of cell physiology [1]. Although early examples were discovered decades ago, large-scale transcriptomic studies have since revealed that mammalian genomes encode thousands of long (>200 nt) transcripts that lack coding capacity, but are otherwise mRNA-like [2-4]. Their biological importance has been controversial, but novel functional lncRNAs with roles, for example, in vertebrate development [5], pluripotency [6] and genome stability [7] are now being described at increasing frequency.

A few recent studies describe interactions between small and long non-coding RNAs, where lncRNAs act either as regulatory targets of microRNA-induced destabilization [8,9] or as molecular decoys of microRNAs [10-13]. Recent results also show that stable circular lncRNAs can bind and inhibit microRNAs [14,15]. Importantly, RNAi-based studies, including silencing of 147 lncRNAs with lentiviral shRNAs [6], show that lncRNAs are, in principle, susceptible to repression by Argonaute-small RNA complexes, despite often localizing to the nucleus. In addition, there are data from crosslinking and immunoprecipitation (CLIP) experiments that support binding of Argonaute proteins to lncRNAs [16,17].

Comparative genomics has revealed that most protein-coding genes are under conserved microRNA control: conserved microRNA target sites are present in 3’ untranslated regions (UTRs) of protein-coding mRNAs at frequencies considerably higher than randomly expected, clearly demonstrating the impact of microRNAs on mRNA evolution [18,19]. While lncRNAs in general are weakly conserved, they may have local patches of strong sequence conservation [20]. It was recently shown that developmental defects caused by knockdown of lncRNAs in zebrafish could be rescued by introduction of putative human orthologs identified based on such short patches [5], supporting that lncRNA functions may be conserved over large evolutionary distances despite limited sequence similarity. It is thus plausible that lncRNAs also have evolved to be targeted by microRNAs despite their overall low conservation, and that this would manifest itself through the presence of target sites in local conserved segments.

### Results

**Figure 1:**
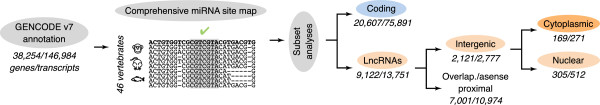
**Workflow to detect conserved microRNA targeting of long non-coding RNAs (lncRNAs).** Conserved microRNA target sites (complementary seed matches) were identified in the GENCODE human gene annotation based on a 46-species multiple sequence alignment as described previously [21]. A total of 1,267 microRNA families were considered. Different subsets of lncRNAs were analyzed for over-representation of sites compared to a random background model.

We used our previously described pipeline to map and assess the evolutionarily conservation of putative microRNA target sites in lncRNAs [21]. Briefly, we mapped complementary matches to established microRNA seed families in the GENCODE v7 lncRNA annotation, which was recently characterized in detail by the ENCODE consortium [4]. Conservation levels were determined based on a 46-vertebrate multiple sequence alignment [22], and sites were scored based on their presence in primates, mammals and non-mammal vertebrates. This allowed us to vary the stringency to consider progressively smaller sets of transcripts with higher conservation levels. We compared observed site frequencies to expected frequencies based on a random dinucleotide model, in protein-coding genes and in subsets of lncRNAs (Figure 1).

**Figure 2:**
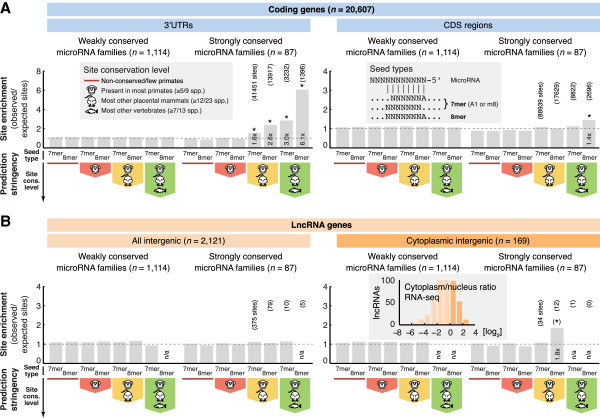
**Ratios between observed and expected microRNA target site frequencies in coding genes and long non-coding RNAs (lncRNAs). (A)** Our methodology was first established on coding genes. The 3’ untranslated regions (UTRs) and coding sequences (CDS) were analyzed separately. We compared observed numbers of seed matches (in parentheses) to randomly expected numbers based on sets of synthetic seeds that preserved the dinucleotide frequencies of the actual seeds. Different prediction stringencies (site conservation level and seed quality) were applied, further explained within gray boxes. The analysis focused on highly conserved microRNA families (*n* = 87), but non-conserved families were included as a control. Bars show mean observed-to-expected ratios from 20 repeated trials. **(B)** Similar analysis based on intergenic lncRNAs and cytoplasmic intergenic lncRNAs. Placental mammal conserved 8-mer sites were present above expectation in a small subset of cytoplasmic intergenic lncRNAs (12 sites for 11 microRNA families, in 8 lncRNA genes). Subcellular localization was determined based on RNA-seq libraries from seven fractionated cell lines. *, empirical *P* <0.05 for ratio being greater than 1; (*), *P* = 0.10; n/a, observed counts to low.

Our analysis revealed widespread presence of conserved target sites in mRNAs, which recapitulates previous observations and establishes our methodology [18,19]. Depending on prediction stringency (conservation level and seed type), seed complementary matches to conserved microRNA families were present at up to 6.1× the expected frequency in 3’ UTRs, and 1.4× in coding regions (Figure 2A). Sites for non-conserved microRNA families, which were included as a negative control, were observed only at expected frequencies (Figure 2A).

Next, we investigated site frequencies in lncRNAs, specifically of the intergenic type to avoid confounding genomic overlaps. In a set of 2,121 intergenic lncRNA genes, we observed no significant enrichment of sites (Figure 2B). Restricting our search to 3’ or 5’ ends of transcripts, or subsets of intergenic lncRNAs previously found to have conserved promoter regions [4], resulted in a similar lack of enrichment (data not shown).

Many described lncRNAs participate in the assembly of riboprotein complexes in the nucleus [1], while microRNAs are considered to be active primarily in the cytoplasm. We used subcellular RNA-seq data to narrow down our analysis to a smaller set of cytoplasmic lncRNAs (*n* = 169), which were also expressed at comparatively high levels (Figure 2B). Pan-mammalian conserved high-quality (8-mer) sites were here observed at 1.8x the expected frequency (*P* = 0.10), which corresponds to a false discovery rate of 56%, but the number of targets and sites was small (12 sites in 8 lncRNA genes, Table 1). One of the eight target lncRNAs (*AC010091.1*) showed distant homology to human protocadherin Fat 4 protein (maximum 36% identity over 94 a.a.), and could thus represent an ancient pseudogene or misclassified coding gene. All others lacked homology to any of 565,000+ known sequences in UniProtKB/Swiss-Prot, and seven out of eight were also classified as long non-coding in a recent RNA-seq-based mapping of human lncRNAs [3].

**Table 1:** Pan-mammalian conserved 8-mer putative microRNA target sites in cytoplasmic intergenic long non-coding RNAs (lncRNAs)

**Target GENCODE**	**Target**	**MicroRNA family**	**Site**	**Site genome**	**Cabili *et al*.**	**UniProtKB/Swiss-Prot**
**ID**	**symbol**		**chromosome**	**position**	**lincRNA**^**a**^	**BLAST**^**b**^
ENSG00000226856.1	*AC093901.1*	miR-182	chr2	118940821	Yes	No hits
ENSG00000231532.1	*AC022311.1*	miR-133abc	chr2	4676715	Yes	No hits
ENSG00000231532.1	*AC022311.1*	miR-22/22-3p	chr2	4676706	↑	↑
ENSG00000231532.1	*AC022311.1*	miR-383	chr2	4676629	↑	↑
ENSG00000233491.2	*AC010091.1*	miR-133abc	chr7	81218260	Yes	E=4e-5(Human FAT4)
ENSG00000233491.2	*AC010091.1*	miR-9/9ab	chr7	81218258	↑	↑
ENSG00000236719.2	*RP11-522D2.1*	miR-30abcdef/30abe-5p/384-5p	chr1	180535222	Yes	No hits
ENSG00000245017.1	*AC013418.2*	miR-138/138ab	chr12	98879829	Yes	No hits
ENSG00000248927.1	*CTD-2334D19.1*	miR-135ab/135a-5p	chr5	120126269	Yes	No hits
ENSG00000248927.1	*CTD-2334D19.1*	miR-19ab	chr5	120126442	↑	↑
ENSG00000250366.1	*AL133167.1*	miR-218/218a	chr14	96389499	Yes	No hits
ENSG00000253507.1	*CTD-2501M5.1*	miR-146ac/146b-5p	chr8	132329800	No	No hits

^a^Annotated as a long non-coding RNA in Cabili MN, Trapnell C *et al*., Genes and Development (2011).

^b^Hits with BLAST E-value <0.5. Repeat masking was performed to avoid matches to, for example, translated SINEs in SwissProt.

Genomic coordinates refer to the Hg19 assembly.

Conserved targeting of lncRNAs by microRNAs is plausible, given that LncRNAs are susceptible to AGO-mediated repression, and that they show patch-wise strong sequence conservation. However, our analysis indicates that this is not a widespread phenomenon, even though a small subset of cytoplasmic transcripts showed a weak enrichment of conserved sites at marginal statistical significance. LncRNAs are currently defined solely based on length and coding capacity, and are as such likely to represent a highly functionally diverse group. It is thus possible that other, not yet defined, subfamilies have evolved to be microRNA targets, but that this signal is too diluted to be detectable in our current analysis.

It should be noted that the GENCODE annotation used here is one of several published lncRNA sets, and while comprehensive, it does not cover all known transcribed loci [3]. Likewise, there are several approaches to target site prediction and detailed results may vary. Notably, our analysis was designed to capture an overall signature of conserved targeting, and when applied to mRNAs it efficiently recapitulated a strong enrichment signal. Different implementations and annotations could give variable results at the level of individual transcripts and sites, but the main conclusion is unlikely to depend on these parameters.

While some established microRNA-lncRNA interaction sites are conserved to various extents, in principle enabling detection by comparative genomics approaches [8-10], others lack conservation despite having experimentally confirmed functions [12,13]. This is consistent with data showing that many non-conserved human microRNA sites can mediate targeting [23]. Notably, even well-characterized lncRNAs, such as *HOTAIR* and *XIST*, have often evolved rapidly, and may show considerable functional and structural differences within the mammalian lineage [24,25]. Our comparative genomics methodology therefore does not exclude that non-conserved and recently evolved targeting could be commonplace, and this motivates further computational and experimental studies.

### Methods

We relied on the GENCODE coding/non-coding classification, and considered as lncRNAs genes that only produced transcripts of the ‘antisense’, ‘lincRNA’, ‘non_coding’ and ‘processed_transcript’ types. We excluded pseudogenes, as well as any gene producing any splice isoform shorter than 200 nt. Genes with symbols corresponding to any RefSeq coding gene, or to the UCSC browser xenoRefGene set, were removed from the long non-coding set, to control for a small number of cases of obvious incorrect coding/non-coding classification in the GENCODE annotation. This resulted in set of 13,751/9,122 lncRNA transcripts/genes. A smaller subset of 2,121/2,777 intergenic lncRNA genes/transcripts were stringently defined by requiring a genomic separation of at least 10 kb to any other annotated gene.

MicroRNA target sites in GENCODE v7 genes were mapped as described previously [21]. Random seed sequences were generated under a dinuclotide model that preserved nucleotide frequencies of the actual microRNA family seeds, and were subsequently mapped in the same way as the actual seed sequences. Ratios of observed-to-expected site counts were calculated based on these random seeds, for different conservation level thresholds and seed match types. To assess the statistical significance of these ratios, 20 sets of random seeds were evaluated, each set being of the same size as the set of actual conserved families (*n* = 87). At least 19/20 cases of ratio >1 were required for significance at the empirical *P ≤*0.05 level, and 18/20 for *P* = 0.10. MicroRNA family definitions and conservation classifications were derived from TargetScan [18]. We used data from a previous study [4] to define subsets of lncRNAs with conserved regulatory regions. The 500 or 250 most conserved intergenic lncRNAs based on either pan-mammal or pan-vertebrate promoter conservation scores (in total, four sets) were analyzed as described above.

RNA-seq data (fastq files) produced within the ENCODE project [26] by the Gingeras laboratory (Cold Spring Harbor Laboratories, Cold Spring Harbor, NY, USA) were obtained through the UCSC FTP server. A total of 1.71 billion 76 nt read pairs from polyA+ nuclear and cytoplasmic fractions from seven human cell lines (Gm12878, HelaS3, HepG2, Huvec, H1hesc, Nhek and K562) were aligned to the human hg19 reference genome with Tophat [27]. The aligner was supplied with GENCODE gene models using the -G option. Genes were quantified using the HTSeq-count utility (http://www-huber.embl.de/users/anders/HTSeq). Cytoplasmic transcripts were defined as having a normalized cytoplasm/nucleus ratio >1. A total of at least 20 mapped reads across all conditions was required, to avoid unreliable cytoplasm/nuclear ratios in the low-abundance range.

Ethical approval or patient consent was not required for this study.

## Abbreviations

CDS: Coding sequence; CLIP: Crosslinking and immunoprecipitation; LncRNA: Long non-coding RNA; UTR: Untranslated region.

## Competing interests

The authors declare that they have no competing interests.

## Author’s contributions

EL designed the study, analyzed data, and wrote the manuscript. BA analyzed data. Both authors read and approved the final manuscript.
